# Supplementary material for: Visualizing the impact of disease-associated mutations on G protein–nucleotide interactions
Source: bioRxiv. 2024 Feb 1:2024.01.30.578006. Preprint. [Version 1] doi: 10.1101/2024.01.30.578006 (PMC10862895; doi:10.1101/2024.01.30.578006)
Supplement: Supplement 1 [file NIHPP2024.01.30.578006v1-supplement-1.pdf]

bioRxiv preprint doi: https://doi.org/10.1101/2024.01.30.578006; this version posted February 1, 2024. The copyright holder for this preprint (which was not certified by peer review) is the author/funder, who has granted bioRxiv a license to display the preprint in perpetuity. It is made available under aCC-BY 4.0 International license.

**Supporting information**

# Visualizing the impact of disease-associated mutations on G protein–nucleotide interactions

Kara Anazia[1,‡], Lucien Koenekoop[2,‡], Guillaume Ferré [1,3], Enzo Petracco[1,4], Hugo Gutiérrez-de-Teran[2 *], Matthew T. Eddy[1,5 *]

[1]Department of Chemistry; University of Florida; Gainesville, FL, 32611; USA

[2]Department of Cell and Molecular Biology, Computational Biology and Bioinformatics, Uppsala University; Uppsala, 75105; Sweden

[3]Present address: Institut de Pharmacologie et de Biologie Structurale (IPBS), Université de Toulouse, CNRS, Université Toulouse III - Paul Sabatier (UT3), Toulouse, France

[4]URD Agro-Biotechnologies Industrielles (ABI), CEBB, AgroParisTech, Pomacle, France

[‡]These authors have contributed equally to this work

[5]Lead contact

*Correspondence: hugo.gutierrez@icm.uu.se, matthew.eddy@ufl.edu

bioRxiv preprint doi: https://doi.org/10.1101/2024.01.30.578006; this version posted February 1, 2024. The copyright holder for this preprint (which was not certified by peer review) is the author/funder, who has granted bioRxiv a license to display the preprint in perpetuity. It is made available under aCC-BY 4.0 International license.

## Supplementary results


| Table of contents | Page # |
|---|---|
| Figure S1. Purification of Gα$_S$ protein and functionality. | S-3 |
| Table S1. Summary of biochemical properties of Gα$_S$ disease-causing variants | S-5 |
| Table S2. Primers designed for Gα$_S$ variants | S-6 |
| Figure S2. Thermal melting profile of Gα$_S$ and diseased variants in GDP and GTPγS determined by circular dichroism. | S-7 |
| Figure S3. Thermal melting profiles of Gα$_S$ and Gα$_S$ variants in the presence of GDP, GTPγS, or with no nucleotide added (apo). | S-8 |
| Figure S4. Backbone root mean square fluctuations (RMSF) values within the switch regions of Gα$_S$ and Gα$_S$ variants in complexes with GDP or GTP | S-9 |
| Figure S5. $^1$H signal assignment in reference spectra of GDP and GppNHp | S-10 |
| Figure S6. Residues closest to bound GTP in the Gα$_S$ nucleotide binding pocket | S-11 |
| Figure S7. Optimization of STD-NMR saturation transfer time | S-12 |
| Figure S8. One-dimensional $^1$H STD-NMR spectra of Gα$_S$ and Gα$_S$ variants in complexes with GDP and GppNHp | S-13 |
| Figure S9. Percent of contact of residues of Gα$_S$ and Gα$_S$ variants with GDP and GTP protons in STD-NMR over MD simulations | S-14 |
| References | S-15 |

bioRxiv preprint doi: https://doi.org/10.1101/2024.01.30.578006; this version posted February 1, 2024. The copyright holder for this preprint (which was not certified by peer review) is the author/funder, who has granted bioRxiv a license to display the preprint in perpetuity. It is made available under aCC-BY 4.0 International license.

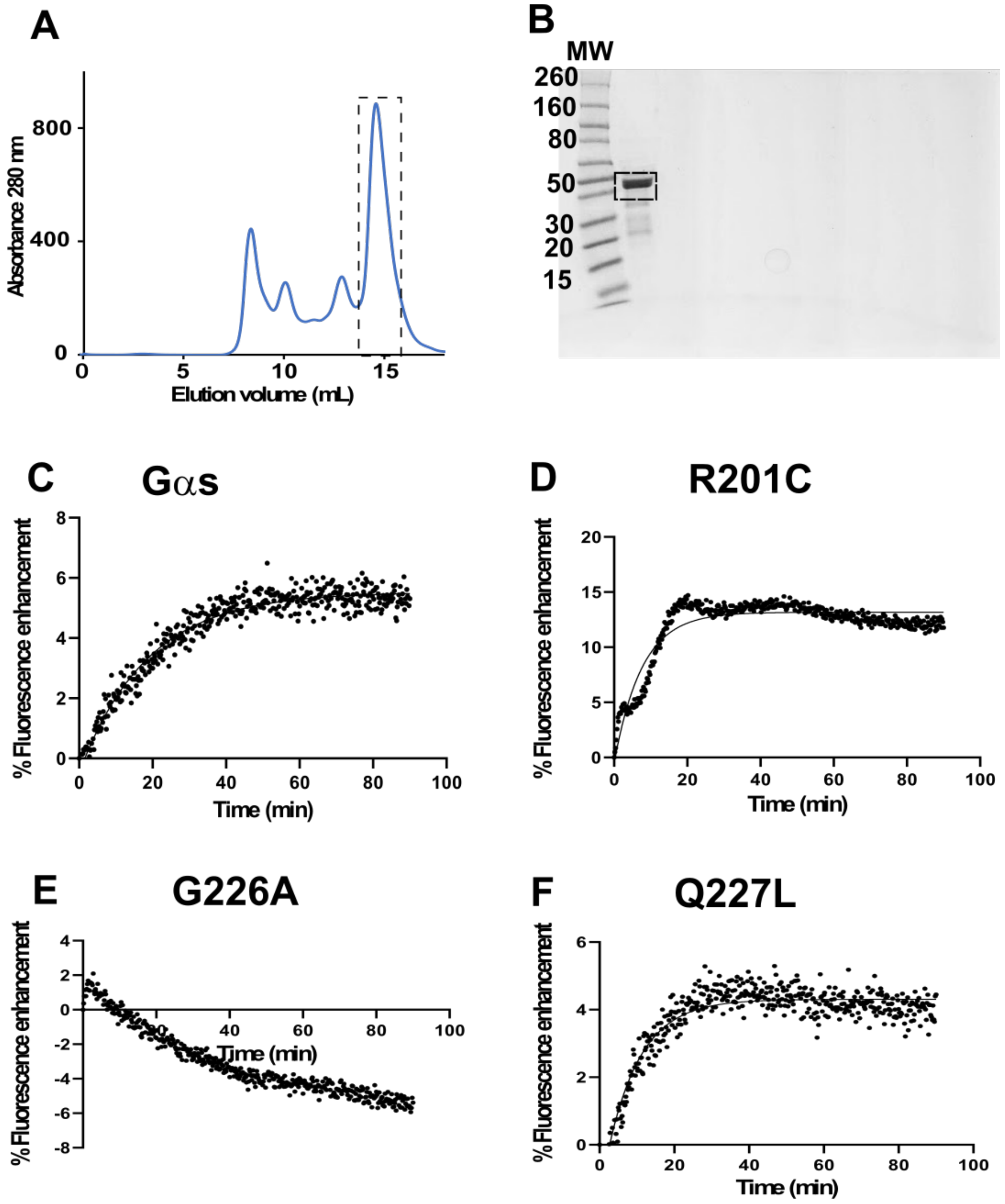


**Figure S1. Purification of Gαs protein and functionality.** *A*, size exclusion chromatogram of Gαs protein purification shown with the boxed peak corresponding to the Gαs protein. *B,* SDS page gel of purified Gαs protein with the molecular weight standard indicating the size of the 44kDa protein. *C,* tryptophan fluorescence assay of Gαs protein showing an increase in tryptophan fluorescence upon GTP binding and G protein activation *D,* tryptophan fluorescence assay of the hyperactivating R201C variant showing a faster increase in tryptophan fluorescence upon GTP binding. *E,*

bioRxiv preprint doi: https://doi.org/10.1101/2024.01.30.578006; this version posted February 1, 2024. The copyright holder for this preprint (which was not certified by peer review) is the author/funder, who has granted bioRxiv a license to display the preprint in perpetuity. It is made available under aCC-BY 4.0 International license.

tryptophan fluorescence assay of G226A variant showing no increase in tryptophan fluorescence upon GTP binding. *F,* tryptophan fluorescence assay of the hyperactivating Q227L variant showing a faster increase in tryptophan fluorescence upon GTP binding.

bioRxiv preprint doi: https://doi.org/10.1101/2024.01.30.578006; this version posted February 1, 2024. The copyright holder for this preprint (which was not certified by peer review) is the author/funder, who has granted bioRxiv a license to display the preprint in perpetuity. It is made available under aCC-BY 4.0 International license.

**Table S1 Summary of biochemical properties of $G\alpha_S$ disease-causing variants**

| $G\alpha_S$ disease variant | Disease presentation | Location in $G\alpha_S$ structure | Activation of adenylyl cyclase | GDP dissociation rate | GTP binding rate | GTPase activity rate | References |
|---|---|---|---|---|---|---|---|
| R201C | Pituitary tumors and lung carcinomas | Switch I | Increased | Decreased | Decreased | Decreased | [1, 2] |
| Q227L | Pituitary tumors | Switch II | Increased | Decreased | Decreased | Decreased | [1, 3] |
| A366S | Testotoxicosis and Pseudohypoparathyroidism | loop connecting β6 and α5 | Increased | Increased | - | Increased | [4, 5] |
| R228C | Pseudohypoparathyroidism | Switch II | Decreased | Increased | Similar to $G\alpha_S$ | Similar to $G\alpha_S$ | [1, 2] |
| R258A | Albright's Hereditary Osteodystrophy | Switch III | Decreased | Increased | Increased | Increased | [2, 6] |
| R265H | Albright's Hereditary Osteodystrophy | Switch III | Decreased | Increased | Increased | Increased | [2] |
| G226A | Lymphoma | Switch II | Decreased | Similar to $G\alpha_S$ | Similar to $G\alpha_S$ | Decreased | [7] |

**Table S1. Summary of biochemical properties of $G\alpha_S$ disease causing variants.** Each variant is characterized in terms of which disease state it is represented in, the location in the $G\alpha_S$ structure, the effect of the mutation on the activation of adenylyl cyclase, the rate of GDP dissociation, the rate of GTP binding and the rate of GTPase activity. The dash indicates that this particular value was not determined within the cited study.

bioRxiv preprint doi: https://doi.org/10.1101/2024.01.30.578006; this version posted February 1, 2024. The copyright holder for this preprint (which was not certified by peer review) is the author/funder, who has granted bioRxiv a license to display the preprint in perpetuity. It is made available under aCC-BY 4.0 International license.

**Table 2. Primers designed for G$\alpha_S$ variants.** List of forward and reverse primers designed to obtain G$\alpha_S$ disease-associated variants.

| Mutation | Directionality | Primer |
|---|---|---|
| R201C | Forward | GACTATGTGCCGAGCGATCAGGACCTGCTTCGCTGCTGCGTCCTGACTTCTGGAATCTTTGAG |
| | Reverse | CTCAAAGATTCCAGAAGTCAGGACGCAGCAGCGAAGCAGGTCCTGATCGCTCGGCACATAGTC |
| G226A | Forward | CAACTTCCACATGTTTGACGTGGGTGCCCAGCGCGATGAACGCCGCAAGTGG |
| | Reverse | CCACTTGCGGCGTTCATCGCGCTGGGCACCCACGTCAAACATGTGGAAGTTG |
| Q227L | Forward | TTCCACATGTTTGACGTGGGTGGCCTGCGCGATGAACGCCGCAAGTGGAT |
| | Reverse | GATCCACTTGCGGCGTTCATCGCGCAGGCCACCCACGTCAAACATGTGGAA |
| R228C | Forward | CACATGTTTGACGTGGGTGGCCAGTGCGATGAACGCCGCAAGTGGATCCAG |
| | Reverse | CTGGATCCACTTGCGGCGTTCATCGCACTGGCCACCCACGTCAAACATGTG |
| R258A | Forward | AGCAGCAGCTACAACATGGTCATCGCCGAGGACAACCAGACCAACCGCCTG |
| | Reverse | CAGGCGGTTGGTCTGGTTGTCCTCGGCGATGACCATGTTGTAGCTGCTGCT |
| R265H | Forward | CATCCGGGAGGACAACCAGACCAACCATCTGCAGGAGGCTCTGAACCTCTTC |
| | Reverse | GAAGAGGTTCAGAGCCTCCTGCAGATGGTTGGTCTGGTTGTCCTCCCGGATG |
| A366S | Forward | CTACTGCTACCCTCATTTCACCTGCTCTGTGGACACTGAGAACATCCGCCGTGTG |
| | Reverse | CACACGGCGGATGTTCTCAGTGTCCACAGAGCAGGTGAAATGAGGGTAGCAGTAG |

bioRxiv preprint doi: https://doi.org/10.1101/2024.01.30.578006; this version posted February 1, 2024. The copyright holder for this preprint (which was not certified by peer review) is the author/funder, who has granted bioRxiv a license to display the preprint in perpetuity. It is made available under aCC-BY 4.0 International license.

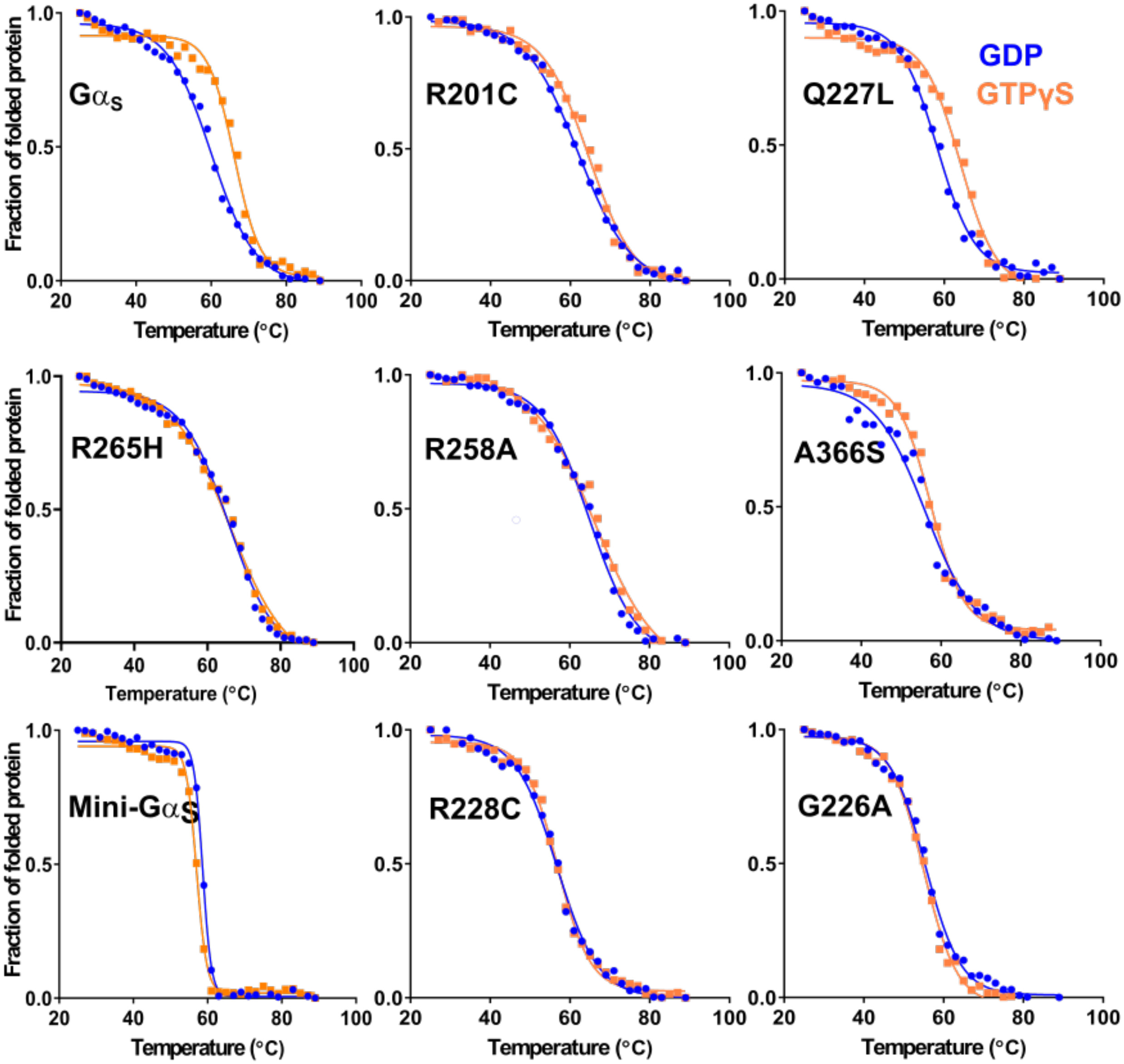


**Figure S2. Thermal melting profile of Gαs and diseased variants in GDP and GTPγS determined by circular dichroism.** The thermal unfolding of Gαs and disease-associated variants bound to GDP and GTPγS monitored by variable temperature single wavelength CD. Same color scheme as Figure 1*A*. The thermal melting temperatures were obtained by fitting the data to a Boltzmann sigmoidal function in GraphPad prism.

bioRxiv preprint doi: https://doi.org/10.1101/2024.01.30.578006; this version posted February 1, 2024. The copyright holder for this preprint (which was not certified by peer review) is the author/funder, who has granted bioRxiv a license to display the preprint in perpetuity. It is made available under aCC-BY 4.0 International license.

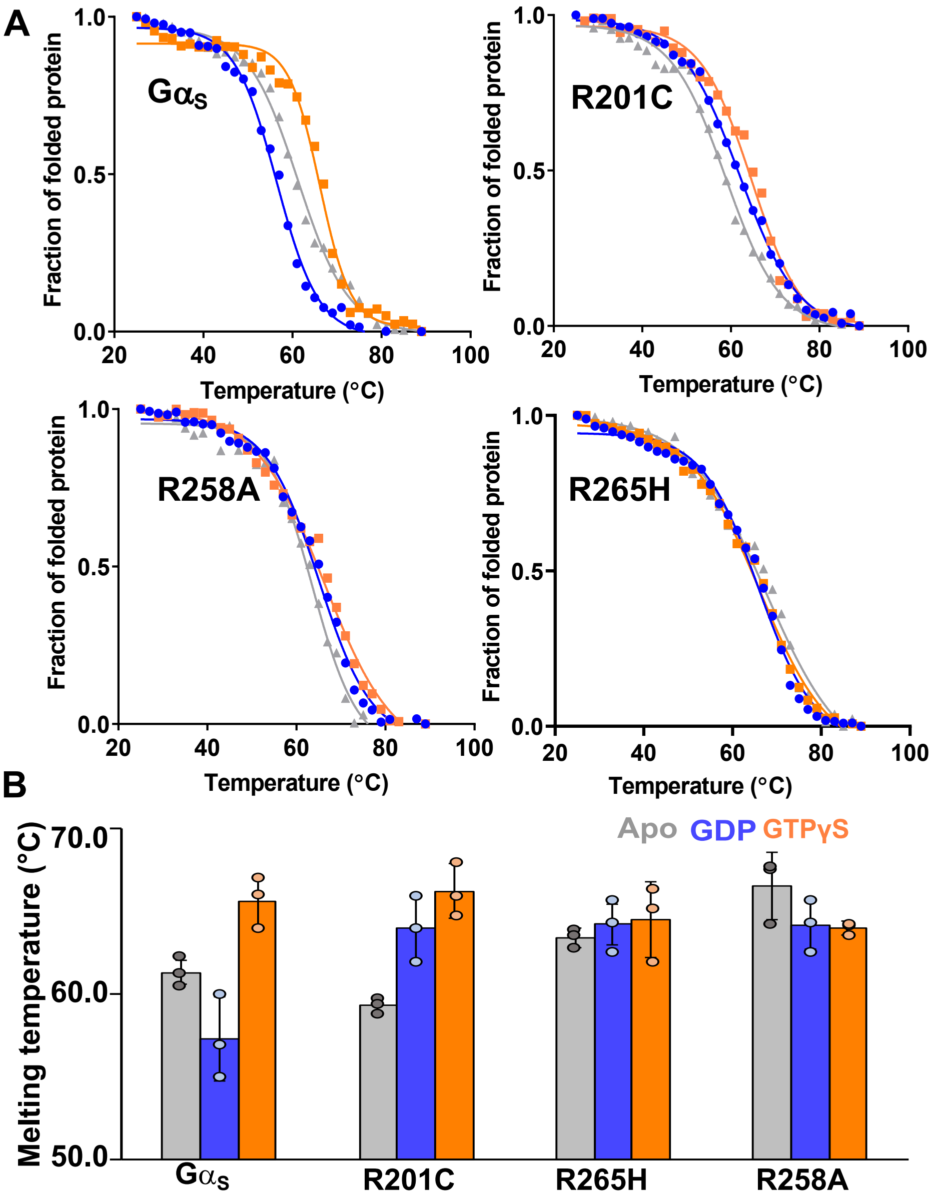


**Figure S3. Thermal melting profiles of Gα$_S$ and Gα$_S$ variants in the presence of GDP, GTPγS, or with no nucleotide added (apo).** *A,* The thermal unfolding of Gα$_S$ and variants Gα$_S$[R201C], Gα$_S$[R258A] and Gα$_S$[R265H] when bound to GDP, GTPγS or with no nucleotide added (apo), as monitored by variable temperature single wavelength CD. Same color scheme used as in Figure 1. *B,* histograms of the melting temperature ($T_m$) values determined by fitting the data shown in panel A. Error bars represent the standard deviation of triplicate measurements.

bioRxiv preprint doi: https://doi.org/10.1101/2024.01.30.578006; this version posted February 1, 2024. The copyright holder for this preprint (which was not certified by peer review) is the author/funder, who has granted bioRxiv a license to display the preprint in perpetuity. It is made available under aCC-BY 4.0 International license.

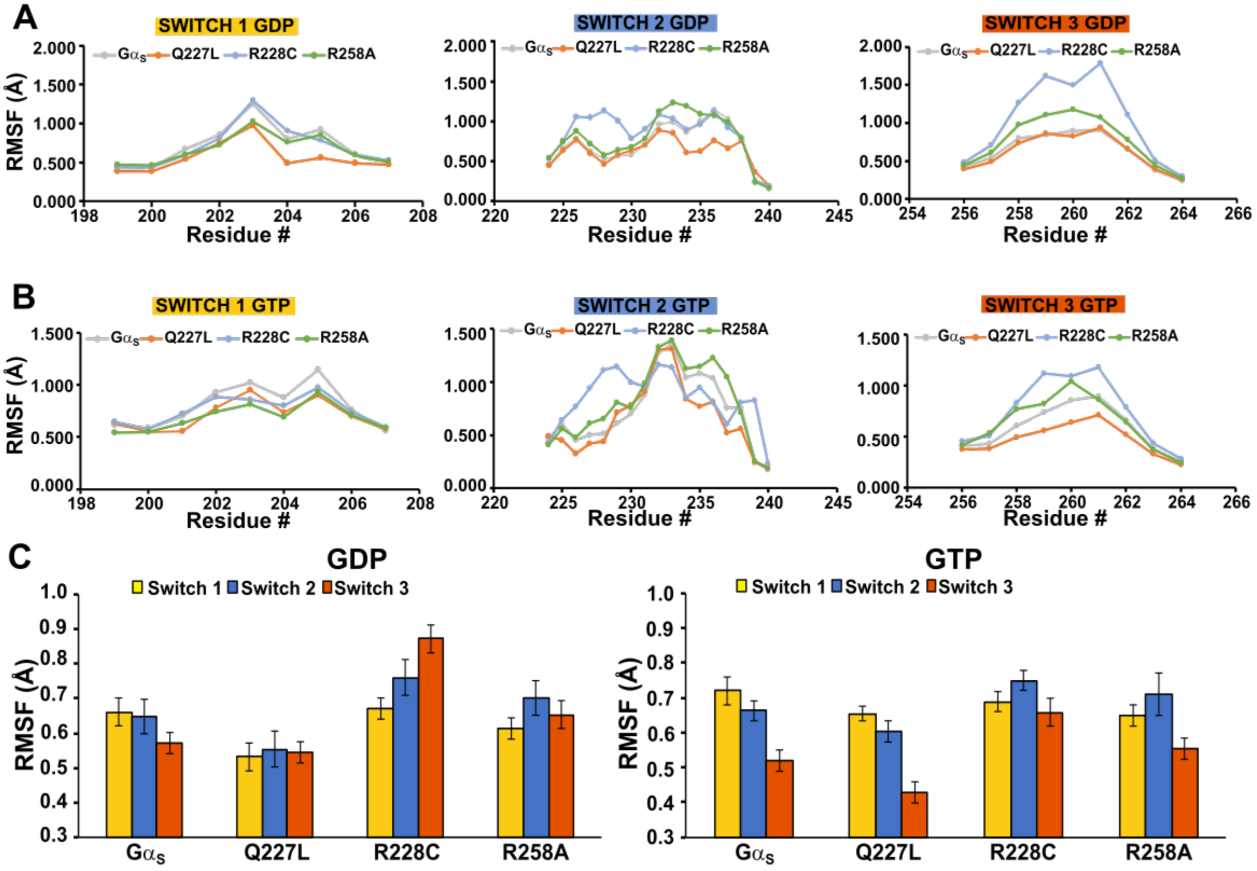


**Figure S4. Backbone root mean square fluctuations (RMSF) values within the switch regions of Gαs and Gαs variants in complexes with GDP or GTP**. *A* and *B,* line plots of backbone RMSF values of residues in Switch I, II and III regions for Gαs (gray), Gαs[Q227L] (orange), Gαs[R228C] (light blue) and Gαs[R258A] (green). *C,* histograms of the average RMSF values for Gαs and the Gαs variants bound to GDP (left panel) and GTP (right panel) in the regions of Switch I (yellow bars), Switch II (blue bars) and Switch III (red bars). Error bars in the histograms represent the standard error of the mean.

bioRxiv preprint doi: https://doi.org/10.1101/2024.01.30.578006; this version posted February 1, 2024. The copyright holder for this preprint (which was not certified by peer review) is the author/funder, who has granted bioRxiv a license to display the preprint in perpetuity. It is made available under aCC-BY 4.0 International license.

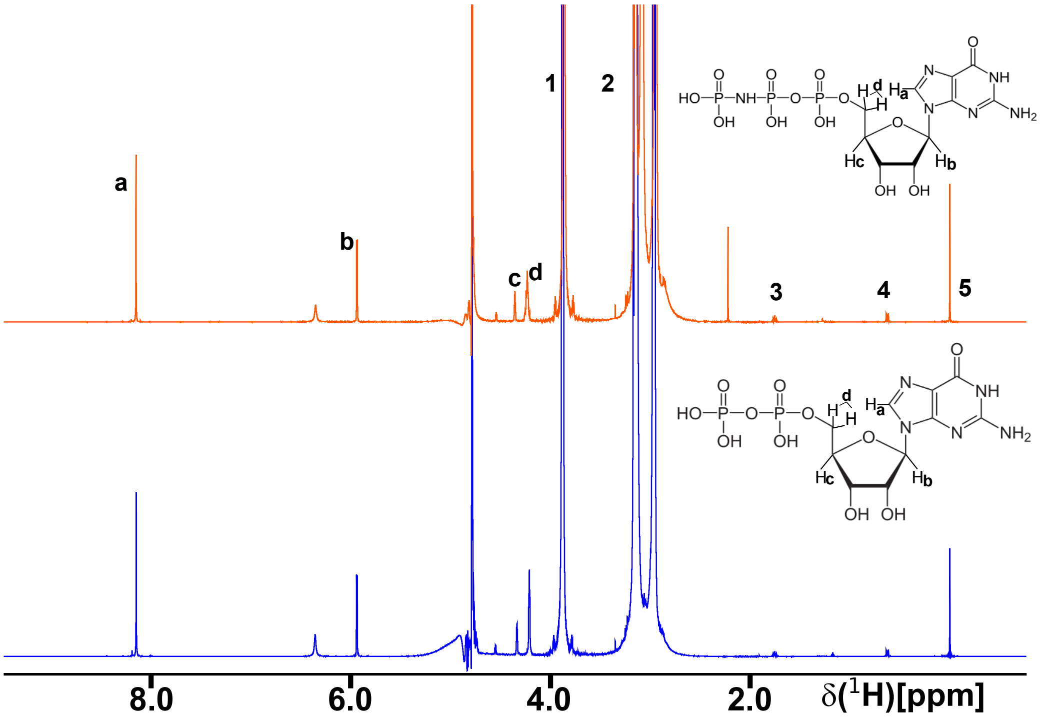


**Figure S5. $^1$H signal assignment in reference spectra of GDP and GppNHp.** 1-dimensional $^1$H-NMR reference spectrum of GDP (blue) and GppNHp (orange) nucleotide. $^1$H signals labeled 'a' through 'd' were utilized in STD-NMR experiments and are shown on the chemical structures of GDP and GppNHp. Assignments were transferred from BMRB entry bmse000270. $^1$H signals labeled 1 and 2 are from HEPES buffer, and $^1$H signals labeled 3-5 are from the sodium trimethylsilylpropanesulfonate (DSS) NMR standard.

bioRxiv preprint doi: https://doi.org/10.1101/2024.01.30.578006; this version posted February 1, 2024. The copyright holder for this preprint (which was not certified by peer review) is the author/funder, who has granted bioRxiv a license to display the preprint in perpetuity. It is made available under aCC-BY 4.0 International license.

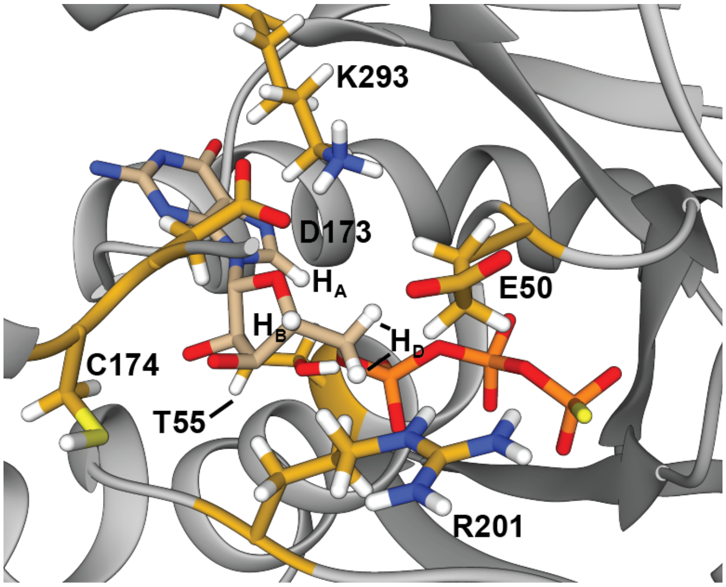


**Figure S6. Residues closest to bound GTP in the G$\alpha_S$ nucleotide binding pocket**. An expanded view is shown of the G$\alpha_S$ nucleotide binding pocket with GTP bound. (shown in tan stick representation, PDB:1AZT) with protons seen in STD-NMR interactions annotated ‘$H_A$’ through ‘$H_D$’. Residues within 6Å of these protons are annotated.

bioRxiv preprint doi: https://doi.org/10.1101/2024.01.30.578006; this version posted February 1, 2024. The copyright holder for this preprint (which was not certified by peer review) is the author/funder, who has granted bioRxiv a license to display the preprint in perpetuity. It is made available under aCC-BY 4.0 International license.

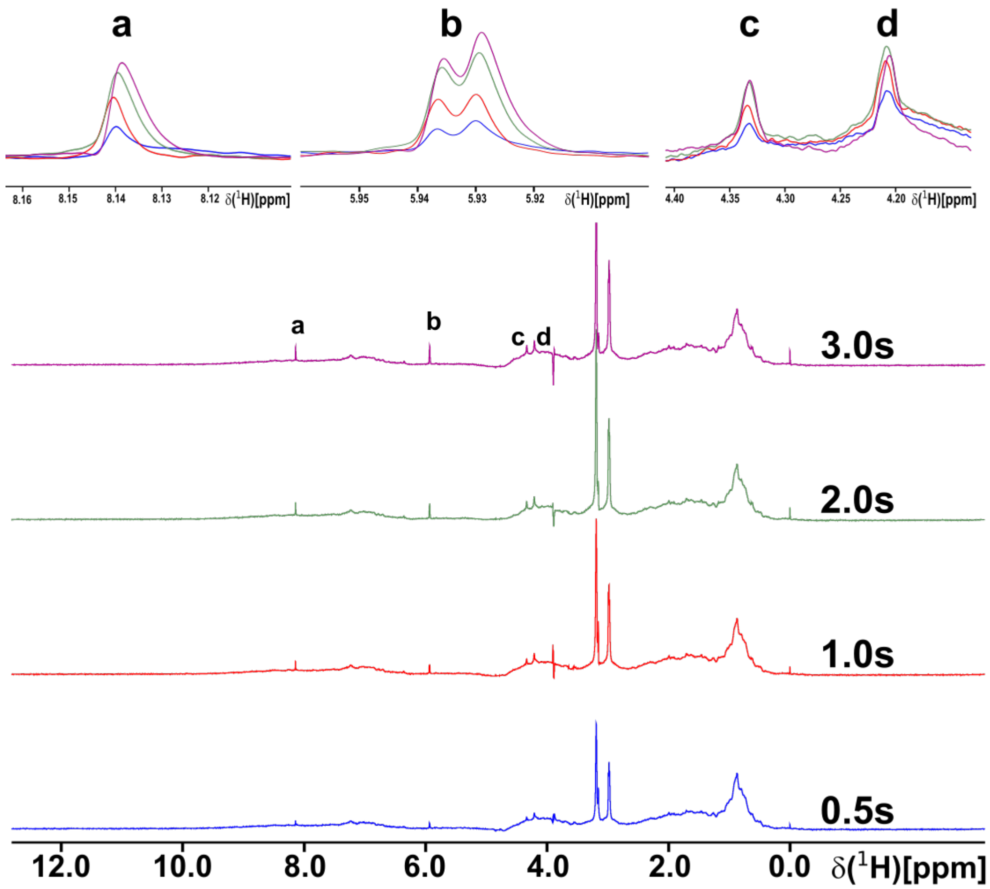


**Figure S7. Optimization of STD-NMR saturation transfer time.** ¹H STD-NMR spectra are shown with 40 μM G$\alpha_S$ and 2 mM GDP recorded with four different saturation transfer times between 0.5 s and 3.0 s. ¹H signals labeled 'a' through 'd' were used to calculate STD-NMR amplification factors. Expanded views of each signal are shown in the panels at the top.

bioRxiv preprint doi: https://doi.org/10.1101/2024.01.30.578006; this version posted February 1, 2024. The copyright holder for this preprint (which was not certified by peer review) is the author/funder, who has granted bioRxiv a license to display the preprint in perpetuity. It is made available under aCC-BY 4.0 International license.

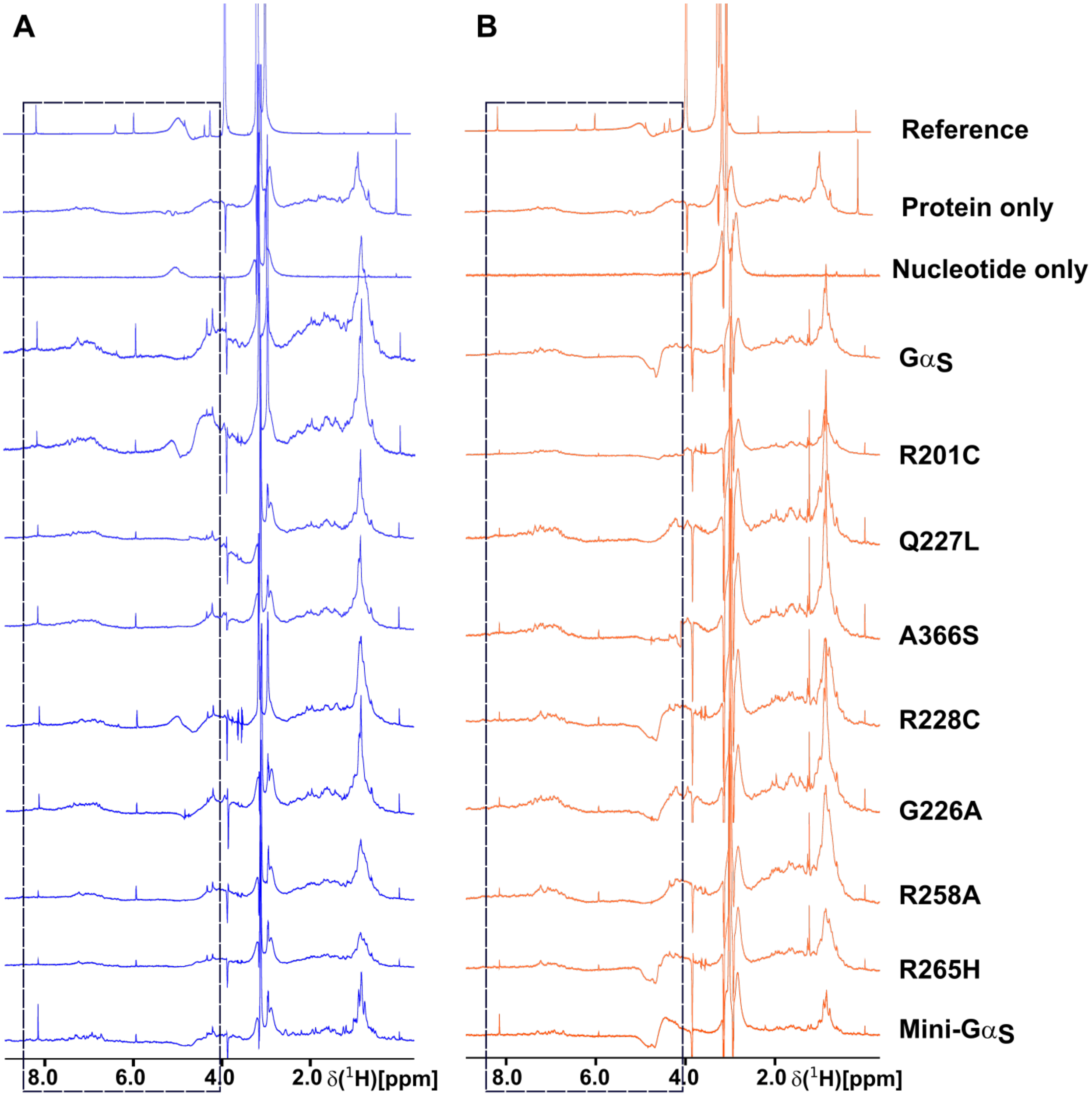


**Figure S8. One-dimensional $^1$H STD-NMR spectra of G$\alpha_S$ and G$\alpha_S$ variants in complexes with GDP and GppNHp**. STD-NMR spectra of G$\alpha_S$ and variants in complex with GDP shown in panel *A* (in blue) and GppNHp shown in panel *B* (in orange). "Reference" is a 1D $^1$H NMR spectrum of GDP, "protein only" is a STD-NMR control experiment with a sample containing G$\alpha_S$ and buffer but no nucleotide, and "nucleotide only" is a STD-NMR control experiment with a sample containing nucleotide and buffer but no protein. Boxed regions indicate areas of interest where an STD-NMR effect is seen.

bioRxiv preprint doi: https://doi.org/10.1101/2024.01.30.578006; this version posted February 1, 2024. The copyright holder for this preprint (which was not certified by peer review) is the author/funder, who has granted bioRxiv a license to display the preprint in perpetuity. It is made available under aCC-BY 4.0 International license.

| Nucleotide proton | Gα$_S$ residue | % GDP contact | % GTP contact |
|---|---|---|---|
| a | G52 | 98.9% | 96.9% |
| | T55 | 0.0% | 2.5% |
| b | D173 | 75.9% | 70.9% |
| | C174 | 0.8% | 2.7% |
| | K293 | 23.3% | 26.4% |
| c | E50 | 18.8% | 28.9% |
| | D173 | 25.7% | 31.4% |
| | R201 | 51.8% | 35.3% |
| | K293 | 2.6% | 4.4% |
| d | E50 | 52.8% | 46.1% |
| | G52 | 0.0% | 17.1% |
| | T55 | 0.0% | 4.3% |
| | D173 | 0.0% | 0.0% |
| | R201 | 45.9% | 32.2% |
| | V202 | 1.3% | 0.0% |
| | L203 | 0.0% | 0.0% |
| | K293 | 0.1% | 0.2% |

| Nucleotide proton | Q227L residue | % GDP contact | % GTP contact |
|---|---|---|---|
| a | G52 | 98.3% | 96.9% |
| | T55 | 1.7% | 2.9% |
| b | D173 | 83.7% | 54.6% |
| | C174 | 0.6% | 1.6% |
| | K293 | 15.7% | 43.8% |
| c | E50 | 96.3% | 10.5% |
| | G52 | 3.4% | 6.8% |
| | R201 | 0.2% | 82.6% |
| | K293 | 0.0% | 0.1% |
| d | E50 | 52.9% | 47.7% |
| | G52 | 1.7% | 8.8% |
| | T55 | 0.0% | 1.9% |
| | D173 | 0.0% | 0.0% |
| | R201 | 45.4% | 41.6% |
| | V202 | 0.0% | 0.0% |
| | L203 | 0.0% | 0.0% |
| | K293 | 0.0% | 0.0% |

| Nucleotide proton | R228C residue | % GDP contact | % GTP contact |
|---|---|---|---|
| a | G52 | 98.3% | 97.3% |
| | T55 | 1.7% | 2.3% |
| b | D173 | 74.0% | 74.0% |
| | C174 | 0.2% | 0.2% |
| | K293 | 25.8% | 25.8% |
| c | E50 | 23.5% | 37.4% |
| | D173 | 29.7% | 18.6% |
| | R201 | 44.5% | 42.1% |
| | K293 | 2.3% | 1.9% |
| d | E50 | 55.7% | 43.9% |
| | G52 | 0.9% | 11.0% |
| | T55 | 0.2% | 0.3% |
| | D173 | 0.0% | 9.3% |
| | R201 | 39.7% | 34.7% |
| | V202 | 2.0% | 0.0% |
| | L203 | 1.5% | 0.0% |
| | K293 | 0.0% | 1.0% |

| Nucleotide proton | R258A residue | % GDP contact | % GTP contact |
|---|---|---|---|
| a | G52 | 98.3% | 94.6% |
| | T55 | 1.6% | 4.9% |
| b | D173 | 62.5% | 40.1% |
| | C174 | 0.4% | 0.2% |
| | K293 | 37.1% | 59.7% |
| c | E50 | 26.8% | 26.2% |
| | D173 | 30.8% | 45.7% |
| | R201 | 41.9% | 25.1% |
| | K293 | 0.5% | 2.9% |
| d | E50 | 53.7% | 47.5% |
| | G52 | 0.0% | 15.1% |
| | T55 | 0.1% | 3.5% |
| | D173 | 0.0% | 0.0% |
| | R201 | 45.4% | 33.7% |
| | V202 | 0.9% | 0.0% |
| | L203 | 0.0% | 0.0% |
| | K293 | 0.1% | 0.3% |

**Figure S9. Percent of contact of residues of Gα$_S$ and Gα$_S$ variants with GDP and GTP protons in STD-NMR over MD simulations.** The percent contact of residues of the Gα$_S$ protein and Gα$_S$[Q227L], Gα$_S$[R228C] and Gα$_S$[R258A] in contact with the protons "a" to d" (d is the average of chemically equivalent protons) in GDP and GTP over the course of MD simulations.

bioRxiv preprint doi: https://doi.org/10.1101/2024.01.30.578006; this version posted February 1, 2024. The copyright holder for this preprint (which was not certified by peer review) is the author/funder, who has granted bioRxiv a license to display the preprint in perpetuity. It is made available under aCC-BY 4.0 International license.

## References


1. O'Hayre, M., et al., *The emerging mutational landscape of G proteins and G-protein-coupled receptors in cancer.* Nature Reviews Cancer, 2013. **13**(6): p. 412-424.
2. Hu, Q. and K.M. Shokat, *Disease-Causing Mutations in the G Protein Gαs Subvert the Roles of GDP and GTP.* Cell, 2018. **173**(5): p. 1254-1264.
3. Graziano, M.P. and A.G. Gilman, *Synthesis in Escherichia coli of GTPase-deficient mutants of* $G_{s\alpha}$*.* Journal of Biological Chemistry, 1989. **264**(26): p. 15475-15482.
4. Taroh Iiri, P.H., Jon M. Nakamoto, Cornelis Van Dop and Henry R. Bourne, *Rapid GDP release from* $G_{s\alpha}$ *in patients with gain and loss of endocrine function.* Nature, 1994. **371**: p. 164-167.
5. Sun, D., et al., *Probing* $G\alpha_{i1}$ *protein activation at single-amino acid resolution.* Nat Struct Mol Biol, 2015. **22**(9): p. 686-694.
6. Warner, D.R. and L.S. Weinstein, *A mutation in the heterotrimeric stimulatory guanine nucleotide binding protein α-subunit with impaired receptor-mediated activation because of elevated GTPase activity.* Proceedings of the National Academy of Sciences of the United States of America, 1999. **96**(8): p. 4268-4272.
7. Lee, E., R. Taussig, and A.G. Gilman, *The G226A mutant of* $G_{s\alpha}$ *highlights the requirement for dissociation of G protein subunits.* Journal of Biological Chemistry, 1992. **267**(2): p. 1212-1218.
